# Comparative Analysis: Potential Barriers to Career Participation by North American Physicians in Global Health

**DOI:** 10.1155/2014/728163

**Published:** 2014-10-27

**Authors:** Daniel S. Rhee, Jennifer E. Heckman, Sae-Rom Chae, Lawrence C. Loh

**Affiliations:** ^1^Department of Pediatric Surgery, Johns Hopkins Hospital, 1800 Orleans Street, Baltimore, MD 21287, USA; ^2^Department of Urology, University of Wisconsin Hospital and Clinics, 600 Highland Avenue, Madison, WI 53792, USA; ^3^Department of Internal Medicine and Pediatrics, University of Illinois at Chicago, Room 1405, 840 S. Wood Street, Chicago, IL 60612, USA; ^4^Dalla Lana School of Public Health, University of Toronto, 155 College Street West, 6th Floor, Toronto, ON, Canada M5T 3M7; ^5^Department of Family Medicine, University of British Columbia, 3rd Floor David Strangway Building, 5950 University Boulevard, Vancouver, BC, Canada V6T 1Z3

## Abstract

Physician interest in global health, particularly among family physicians, is reflected by an increasing proliferation of field training and service experiences. However, translating initial training involvement into a defined and sustainable global health career remains difficult and beset by numerous barriers. Existing global health literature has largely examined training experiences and related ethical considerations while neglecting the role of career development in global health. To explore this, this paper extrapolates potential barriers to global health career involvement from existing literature and compares these to salary and skills requirements for archetypal physician positions in global health, presenting a framework of possible barriers to sustained physician participation in global health work. Notable barriers identified include financial limitations, scheduling conflicts, security/family concerns, skills limitations, limited awareness of opportunities, and specialty choice, with family practice often closely aligned with global health experience. Proposed solutions include financial support, protected time, family relocation support, and additional training. This framework delineates barriers to career involvement in global health by physicians. Further research regarding these barriers as well as potential solutions may help direct policy and initiatives to better utilize physicians, particularly family physicians, as a valuable global health human resource.

## 1. Introduction

Unprecedented interest in global health among physicians in training has driven calls for more opportunities [1] and an expansion of global health experiences into medical school and residency program curricula [2, 3]. Data from the Association of American Medical Colleges (AAMC) found that, from 2006 to 2010, nearly 30% of graduating medical students participated in global health experiences during their medical school training [4]. These experiences commonly take the form of research or clinical experiences abroad, often in partnership with institutions in the host country [5–8], with literature increasingly investigating the practical and ethical considerations surrounding the participation of medical students and resident physicians in global health experiences [1, 3, 9, 10].

Following participation in global health experiences, medical trainees and new physicians often express a desire for continued career involvement in global health work abroad. This interest is particularly pronounced among young family physicians, as demonstrated in studies that reveal an association between trainee interest in global health and entry into family practice and/or primary care [11–15]. However, published literature remains sparse regarding sustained career involvement by physicians in global health efforts.

This research attempts to address this gap by comparing potential barriers described in the existing literature with the realities of global health career involvement. We use published barriers to physician participation in global health experiences as a proxy for long-term career barriers and compare them with contextual data from various archetypal global health positions. The subsequent framework forms the basis for further research into the nature of career involvement by physicians in global health work.

## 2. Methods

Our paper represents a comparative analysis between extrapolated barriers to global health careers from existing literature about trainees and published requirements for career opportunities with major global health organizations and agencies. We conducted a literature search in PubMed, combining concept searches using keyword and MeSH headings for global health with a similar search for training experiences, specialization, and career development. Abstracts from this nested search were subsequently examined individually for relevance, and germane articles were then reviewed to identify any described barriers to participating in global health experiences and solutions to these barriers for inclusion in the final framework.

We also searched papers and expert opinion in grey literature to identify potential career opportunities in global health classically pursued by physicians. After identifying a few well-known examples of major organizations in different career opportunity categories, we then examined the job requirements, salaries, and skills listed for physician positions posted by these organizations to validate the extrapolated barriers. This corroboration formed the basis of our subsequent discussion and analysis.

## 3. Results

### 3.1. Opportunities and Context

Physician participation in global health is not novel, but the nature of modern travel and communication has made the possibility of long-term career involvement seem less elusive for a new generation of physicians. At the same time, broad and diverse definitions of “global health” have led to heterogeneity of opportunities pursued by physicians [16, 17]. These varied possibilities extend beyond the traditional provision of clinical care to underserved communities overseas into work in research, public health, international development, education, and humanitarian assistance. Less traditional physician roles may include international development or public health efforts with intergovernmental organizations (IGOs), nongovernmental organizations (NGOs), and governmental organizations, roles within the private sector, or academic roles in education or faculty development. With some definitions of global health, physician involvement even extends to incorporate work in their home communities, providing care or conducting research among vulnerable populations, though this is less often termed “global health work” in either a work or an educational context and was not included in our examination.

Given their portability, flexibility of scheduling, and broad skill set, family physicians involved in primary care are often able to enter such roles with relative ease compared to other physician specialties. However, they can face similar barriers to doing so, given the demands of contemporary medical training and practice, the precarious nature of many global health jobs, and limited career development and mentorship through a complicated array of career entry points. Consequently, both primary care and specialty physicians experience difficulty in advancing and sustaining global health involvement in their careers.

Examining the current literature using these themes mostly identifies involvement of trainees in short-term educational experiences. Comparing the published barriers to participation in short-term educational experiences to employment requirements for various global health positions identifies potential barriers which might limit wider career involvement by physicians in global health. These include limited financial compensation, time and scheduling constraints, lack of global health education or skills, personal/family commitments, security concerns, differences in opportunities by specialty, and ethical considerations [18]. Our comparative analysis of the literature also identified a number of potential solutions to these proposed barriers, listed in Table 1.

### 3.2. Barriers and Solutions

#### 3.2.1. Finances

A survey of medical students in the USA found that students who had participated in a global health experience hoped to “incorporate [global health work] into their practice, despite heavy debt loads. However, financial obligations required [respondents] to maintain the level of income generated in a U.S.-based practice” [11]. These obligations include the repayment of student loans, requirements for self-financing of opportunities, and limited remuneration for both global health positions and the funding of associated research. Surveys of residents in the USA and Canada found that nearly 50% of respondents had over $100,000 in educational indebtedness [19], with residents and fellows identifying financial issues as a primary barrier to participating in global health work abroad [11, 20].

This barrier is corroborated through comparing these concerns and debt loads against the standards of remuneration offered for work in global health service and research. Considering the archetypical examples of Doctors Without Borders or the World Health Organization, one finds that the former provides a monthly salary of US$1,404 plus a small per diem, accommodation, and insurance coverage, while entry level positions for the latter provide $66,000 per annum [21]. A position as an overseas medical officer with the Centers for Disease Control and Prevention can provide an annual starting salary between $72, 390 and $130,800. This contrasts with the average annual compensation for practicing physicians in the USA, which is notably higher: $174,000 for family practice, $188,000 for internal medicine, $272,000 for emergency medicine, $295,000 for general surgery, $348,000 for urology, and over $400,000 for orthopedic surgery [22]. Additionally, pursuing global health work abroad through academic teaching or research is further influenced by the well-known remuneration disparity that exists compared to community practice. Interest in research can require additional skills and personal commitment to secure temporal funding in the form of grants, a difficult option in the face of competing day-to-day clinical demands and professional responsibilities.

One potential solution to mitigate financial barriers lies in loan forgiveness programs for those serving in underserved, low-income countries. An International Health Service Corps for US health professionals was first considered as early as 1987 [23]. A 2005 report by the Institute of Medicine revisited this idea, recommending the establishment of a federally funded US Global Health Service. Through awarding fellowships and providing partial repayment of student loans, such a program would send midcareer professionals overseas to augment local responses to global health issues. Such a program could foster partnerships and create a global health employment clearinghouse for paid or volunteer positions [24].

This idea was eventually detailed in a commentary by Kerry et al. and formed the basis for the development of the independent, not-for-profit Global Health Service Partnership, a collaboration between Seed Global Health and the Peace Corps with technical support from PEPFAR [13]. This voluntary program addresses financial considerations by providing $30,000 of loan repayment per year of service along with travel, room, and board [25]. The program also includes other structural elements (e.g., work in multidisciplinary teams directed by local leadership) that address other barriers described below. While evaluation of program outcomes is pending, the strategy represents one potential model by which some physicians, otherwise limited by debt, could be assisted in incorporating global health into their career.

#### 3.2.2. Time and Scheduling Conflicts

Residents surveyed in one study also identified “training obligations and staffing needs” as “significant” and noted that obtaining credit for international rotations was a struggle. Many respondents described having to sacrifice their limited vacation time [19]. The Canadian survey also had 54% of respondents identify “lack of elective time” as a major concern [20]. The enormous amount of clinical experience required during training limits the amount of time that can be spent on electives abroad, which can prevent physician trainees from acquiring international health experience and training [21, 26, 27]. In addition, for practicing physicians, obligations to an academic department or to a private group could limit time allowed away. Then, global health work must be self-financed and mainly occur during vacation time [20].

Practicing physicians will continue to relegate time spent on global health work to vacation or spare time (for institutional physicians) or unpaid time for independent family physicians, unless some degree of compensation can be arranged for these efforts (i.e., contracting out for time, incorporating into one's work hours). Solutions to time and scheduling conflicts are thus tightly intertwined with financial barriers and can potentially be resolved through institutional commitments toward protecting paid time for global health responsibilities while supporting physicians to undertake secondments or sabbaticals with global health organizations or partner healthcare institutions in low-resource settings.

#### 3.2.3. Lack of Relevant Education or Skills

Global health perspectives and competencies are not typically acquired through conventional medical education. Given the community-oriented nature of global health work, population-based intervention strategies and public health skill sets are extremely useful. However, only 30% of US medical schools offer any training or counseling for students prior to departing for global health experiences; thus, many students and residents go unprepared which limits their efficiency and effectiveness [28]. Working as a clinician-educator in a resource-poor setting may be significantly different from practice during training, requiring a different set of skills in cultural sensitivity, emotional intelligence, and ethical behavior in the setting of extremely challenging circumstances. Lack of these skills could also perpetuate some avoidable adverse effects on the community being served. Exposure to global health competencies could go a long way in preventing unnecessary harm.

Drawing from the lack of skills associated with short-term experiences, there is growing interest among physicians to pursue advanced degrees or certificates to gain perceived “essential” global health competencies. Commonly, this involves completion of a Master of Public Health (MPH) degree, though other physicians might undertake a Diploma in Tropical Medicine and Hygiene (DTM&H) or undergo various short courses in global health. Pursuing such training represents an additional investment of time and finances, compounding the already heavy demands of early training.

Medical schools are beginning to address this barrier by adopting competencies in global health as part of their standard curriculum. One taskforce suggested course designs that addressed subjects such as the global burden of disease, socioeconomic and environmental determinants of health, health systems, global health governance, human rights and ethics, and cultural diversity and health [29]. Such curricula may prove essential in beginning to address the knowledge barrier to global health career involvement.

Many examples of medical schools and residencies that have formally integrated global and population health into clinical training exist [30, 31]. At the same time, global health-focused residencies and fellowships have begun to provide designated training in health disparities, cultural competency, public health, research, and health systems to interested trainees, often with significant time spent at international sites and a heavy emphasis on education and capacity-building. Their ultimate effects on sustained, responsible career involvement in global health will require careful evaluation [32, 33].

#### 3.2.4. Safety

Many global health elective programs for medical students and residents lack formal structure, which means that individuals are responsible for the safety aspect of their own experience. Additionally, countries with the greatest need are, at times, the areas where safety is of grave concern to those desiring to work in these regions. In 2008, 260 aid workers, the highest annual toll on record, were killed, kidnapped, or seriously injured while in their operational settings. Though the majority of these attacks took place in three countries (Somalia, Afghanistan, and Sudan), the deterioration of safety in international contexts is evident [34]. While groups such as Physicians for Human Rights have actively voiced condemnation against attacks on healthcare workers, the amnesty previously assumed for healthcare workers is no longer universal, presenting an additional, potentially substantial barrier for those desiring to pursue careers abroad.

Risks to personal health can also be considerable in the age of dengue fever, Ebola, and MERS outbreaks, particularly in the context of resource-poor communities. Additionally, instability and deficiencies in infrastructure, healthcare workers, and supplies can make obtaining adequate urgent or routine medical care in a timely manner for oneself or one's family very difficult.

The risks of working in high-risk regions will naturally remain a major deterrent for otherwise eager physicians. However, a widened awareness of options for global health work that could be performed domestically such as research, policy or program development, academic partnerships, and health systems strengthening represent alternative paths that could make a significant impact in these areas.

#### 3.2.5. Personal/Family Commitments

Family commitments were also described as a barrier to global health involvement, particularly to field work abroad. 36% of resident physicians surveyed by Powell et al. identified family concerns as an obstacle to participation in global health opportunities [35]. Commitments to family also figured prominently in the surveys of surgical residents [19]. Family commitments involve significant amounts of financial support and time, and partners and children may not be as mobile as a physician pursuing work abroad. Additionally, partners may face sacrifice of their own career goals and difficulty transitioning to a new way of life.

One benefit of short-term opportunities might be to assess the family's response to work abroad and consider whether longer absences could be manageable [33]. This suggests that providing opportunities for families to experience life abroad, as well as possible relocation assistance for spouses, could help to mitigate this barrier for those seeking longer-term involvement in global health. As mentioned for safety concerns, increasing awareness of the wide range of alternative global health career paths beyond clinical work abroad would be an excellent alternative for those with family commitments precluding foreign travel.

#### 3.2.6. Specialty Choice

The relationship between global health and public health may put some physician specialties at an inherent disadvantage. Family medicine, for example, lends itself easily to global health work, while other hospital-dependent specialties (e.g., surgery) are further removed from “getting started” and finding the right opportunities may be difficult. One study found that “many institutions in the United States have well-established international programs in* non-surgical* graduate disciplines, such as internal medicine, pediatrics, and family practice” [36]. Surgical disciplines traditionally have approached global health through voluntary surgical trips, which require extensive mobilization of resources. More recently, interest in surgical disciplines has extended beyond volunteer medical service to strengthening health systems with the goal shifting from “How many people can we help in a short period of time?” to “How can we lay the groundwork for a sustainable solution for the community?” This represents a valuable potential paradigm shift that may both mitigate this barrier and improve impact.

Radiology is another nontraditional field that is making significant strides to address global health concerns. Organizations such as RAD-AID and Imaging the World have pioneered programs to help provide low-cost, high-quality imaging modalities and the training of local health workers to utilize them. Providing even basic radiologic imaging such as ultrasound and X-rays in conjunction with the teaching of local providers in their use and interpretation can improve patient care significantly. Not only can it provide more reliable diagnosis, but it aids in determining whether urgent referral to a higher-level care center is needed versus observation or care at a lower-level care facility. Imaging can also encourage community participation; obstetrical ultrasound imaging has been shown to bolster antenatal visit participation and improve maternal and neonatal outcomes. On a population level, these programs can save resources and, more importantly, reduce significant morbidity and mortality.

Global health and development efforts increasingly recognize that upstream, systems-level interventions have the greatest promise for lasting impact. Following this example, physicians in fields traditionally not considered to have a strong presence in global health can progress their field's presence through the development of sustainable, population-level programs to address the existing needs.

#### 3.2.7. Ethical Considerations

Working in global health has unique ethical considerations in addition to those faced in domestic practice. Patients in the developing world who are often marginalized, impoverished, and faced with many health threats may be vulnerable to exploitation due to power imbalances in the physician/patient relationship [37]. Some physicians initially interested in working abroad or in global health may forgo global health career involvement due to justifiable concerns surrounding the appropriateness of an intervention or research, their ability to provide quality care, and the appropriate use of resources. Similarly, short-term medical experiences often place disproportionate burdens on the partner site, consuming limited resources in the host community.

The development and application of ethical codes is a first step to addressing this barrier. Wilson and others describe four guiding principles for global health involvement, including service, sustainability, professionalism, and safety [10]. Collaborations such as the Working Group on Ethics Guidelines for Global Health Training have also formed guidelines of best practices for field-based global health experiences for sending and host institutions, trainees, and sponsors [38]. Additionally, case-based curricula are being increasingly developed and used to prepare trainees before embarking on field experiences [39]; completing such training has wider implications for sustained career practice. Though the impact on the ground is difficult to measure, acquiring a greater familiarity with real-life experiences can serve as an appropriate introduction to building a foundation in global health ethics. Together with formal education at the start and throughout a global health career, thoughtful and persistent attention to ethical practice can help to optimize the physician's experience and the positive impact on the host community.

## 4. Discussion

Global health experiences are widely sought after by young physicians and family physicians. Literature shows that participants in early career experiences are more likely to pursue family medicine and primary care as a career and often remain interested in incorporating global health into their future career. Notably, most barriers are modifiable, and the published literature does not specifically address nonmodifiable characteristics and their potential role as a barrier to global health participation. Meanwhile, parallel literature shows the influence of characteristics on overall specialty choice (e.g., gender, socioeconomic or demographic status, or academic/community based nature of training) [40].

These barriers can limit the ability of young physicians to translate global health interest into effective career involvement, and many motivated individuals will decide not to participate in global health work or undertake such work in limited measure with similarly limited impact. Mitigating identified barriers could thus open doors to a huge resource of physicians who might then dedicate their careers to global health.

Solutions to these barriers will prove more challenging. This generation of physicians has demonstrated a notable increase in interest in global health, creating a demand for more opportunities. While interest may arise on individual levels, it will be critical for institutions and governments to develop overarching plans to optimize global health human resource planning to match interest with stable, meaningful opportunities. At the same time, the balance between devoting resources to bringing physicians from the Global North to the Global South and using these resources to strengthen the training of local providers and increase the capacity of local systems continues to be a critical health human resource topic that must be explored and understood. Ongoing planning will require more than simple identification and publication of opportunities but is closely linked to ongoing work to develop consistent physician standards and competencies in global health being undertaken in many major industrialized countries, while simultaneously strengthening local health systems in the Global South.

From these standards, clear career pathways (e.g., assessment and certification via training and alternative pathways, financial support) can address barriers while ensuring consistency among global health physician skill sets. Further, given the multidisciplinary nature of global health work, any human resource planning and training development must also recognize the crucial role of nonphysician professionals in mitigating some of these identified barriers. Most importantly, any future policies and evaluation must consider the potential impacts that could be exerted on populations in the Global South through increasing career participation in work abroad by providers from the Global North. Any policy or program must keep the health outcomes of communities abroad as their primary focus, ensuring that physician participation is a net positive, rather than a burden, on local health systems and partners.

Irrespective of the form any solutions take, it is important to reiterate the key message of our review: the need and desire to participate are evident and the barriers and deficiencies are clear. Future research, policy, and programmatic efforts are needed to secure physicians who may valuably serve in global health efforts. The creation of appropriate national interest groups would also support efforts to develop core competencies, mitigate barriers, and foster shared values (e.g., focusing on outcomes for populations in the Global South, advocating for human rights and health equity, and supporting simultaneous efforts to strengthen local provider knowledge and systems). Family physicians, given their generalist skill set, community orientation, and interest in social justice issues, are well positioned to be at the forefront of any effort to better justify and support the sustained involvement of physicians in global health efforts.

## Figures and Tables

**Table 1:** Derived potential barriers to physician career involvement in global health work and potential solutions.

Barriers	Solutions
(i) Finances	(i) Loan forgiveness programs

(i) Time and scheduling conflicts	(i) Increased elective time

(i) Inadequate global health training/competencies	(i) Incorporation of global health education into medical school curricula and training programs

(i) Safety concerns	(i) Heightened awareness (ii) Alternative options for involvement

(i) Personal and family commitments	(i) Family support (ii) Alternative options for involvement

(i) Specialty choice	(i) Redefine specialty approach to global health (ii) Increase partnerships (iii) Practice more system-based care

(i) Ethical considerations	(i) Establish guiding principles for global health involvement
